# Transduction and Genome Editing of the Heart with Adeno-Associated Viral Vectors Loaded onto Electrospun Polydioxanone Nonwoven Fabrics

**DOI:** 10.3390/biom14040506

**Published:** 2024-04-22

**Authors:** Kotoko Furuno, Keiichiro Suzuki, Shinji Sakai

**Affiliations:** 1 Department of Materials Engineering Science, Graduate School of Engineering Science, Osaka University, 1-3 Machikaneyama-cho, Toyonaka 560-8531, Japan; u552610g@alumni.osaka-u.ac.jp; 2 Institute for Advanced Co-Creation Studies, Osaka University, 1-3 Machikaneyama-cho, Toyonaka 560-8531, Japan; 3 Graduate School of Frontier Bioscience, Osaka University, 1-3 Yamadaoka, Suita 565-0871, Japan

**Keywords:** polydioxanone, electrospinning, gene delivery, genome editing, electrospun nonwoven fabrics

## Abstract

In this study, we introduce electrospun polydioxanone (PDO) nonwoven fabrics as a platform for the delivery of adeno-associated virus (AAV) vectors for transduction and genome editing by adhering them to organ surfaces, including the heart. AAV vectors were loaded onto the PDO fabrics by soaking the fabrics in a solution containing AAV vectors. In vitro, the amount of AAV vectors loaded onto the fabrics could be adjusted by changing their concentration in the solution, and the number of cells expressing the green fluorescent protein (GFP) encoded by the AAV vectors increased in correlation with the increasing amount of loaded AAV vectors. In vivo, both transduction and genome editing resulted in the observation of GFP expression around AAV vector-loaded PDO fabrics attached to the surfaces of mouse hearts, indicating effective transduction and expression at the target site. These results demonstrate the great potential of electrospun PDO nonwoven fabrics carrying therapeutic AAV vectors for gene therapy.

## 1. Introduction

Polydioxanone (PDO), produced by the ring-opening polymerization of p-dioxanone monomers, is a synthetic, absorbable polymer. The polymerization method allows tight control over the molecular weight and physical properties of PDO. Notably, PDO is valued for its biocompatibility and ability to degrade gradually, making it predominantly used for medical sutures. Distinct from other bioresorbable polymers, like poly-L-lactic acid and polyglycolic acid, which find use in medical scenarios, PDO uniquely combines elasticity, flexibility, strength, and a more extended degradation period. These properties make PDO an exceptionally versatile material for various biomedical applications, including drug delivery systems and tissue engineering [1].

One of the products made from PDO for these applications is electrospun fibers [2,3,4]. Miele et al. reported the usefulness of nonwoven fabrics of electrospun PDO fibers in skin tissue engineering [3], while Goonoo et al. presented the application of the fabrics in skeletal tissue engineering [5]. Electrospinning transforms a polymer solution into fine fibers using an electric field. The fabrics consisting of fine fibers have features such as an improved performance due to the individual fiber size, surface/interface effects, and superior optical, electrical, mechanical, and biological properties [2]. The elasticity of PDO ensures that the fabric adheres to and moves with the pulsating surfaces of the target tissues and organs. In addition, the shape of the nonwoven fabric allows it to be easily applied directly to the surfaces of the target organs during surgery, and it can be adjusted as needed to fit various organ sizes and shapes.

Gene delivery is a promising approach for the treatment of heart failure [6,7,8], a major global health problem responsible for significant morbidity and mortality. Heart failure remains a leading cause of mortality worldwide, with millions of individuals affected and a significant burden on healthcare systems. Improving the methods of gene delivery to the heart has the potential to revolutionize treatment protocols and significantly reduce the morbidity and mortality associated with this condition. Therapeutic genes involved in such treatments are typically delivered using viral vectors, including lentivirus, adenovirus, and adeno-associated virus (AAV) vectors [9]. Notably, AAV vectors are non-pathogenic and produced in systems without helper viruses, making them safer for gene therapy [9]. Their high efficiency in transducing both dividing and non-dividing cells is attributed to the distinct tissue tropism determined by the serotype of the AAV vector [10,11,12]. The gene transduction efficiency, a critical measure in gene therapy, refers to the ability of a vector to introduce therapeutic genes into host cells effectively. High efficiency is paramount for the success of gene therapies in treating diseases, and choosing the vector and delivery methods is a crucial component of therapeutic design. In addition, AAV vectors are capable of maintaining gene expression for prolonged periods and exhibit lower immunogenic responses compared to other viral vectors, making them an attractive option for gene therapy in the treatment of heart failure [9,13].

In recent years, the field of genome editing, encompassing the manipulation of target genes within the genome, has witnessed rapid advancements with the introduction of various artificial DNA nucleases, including CRISPR-Cas9. This progress facilitates the selective modification of genome sequences across a broad spectrum of cells and organisms. In essence, the induced DNA double-strand breaks by artificial DNA nucleases are primarily repaired through homology-directed repair (HDR) or non-homologous end-joining (NHEJ) pathways, leading to targeted gene knock-ins or knockouts, respectively. Significantly, the conventional HDR-mediated gene knock-in method, capable of modifying target sequences to any desired sequence, relies on DNA repair mechanisms associated with the cell cycle. This approach is limited to actively dividing cells, presenting challenges in living organisms primarily composed of non-dividing cells wherein the cell cycle is arrested. The previously developed Homology-Independent Targeted Integration (HITI) method, pioneered by us, represents a groundbreaking knock-in technique relying on NHEJ independently of the cell’s division state [11]. This innovation enables gene knock-ins in various non-dividing tissues and organs, including the brains and hearts of live mice. This success stands in contrast to conventional HDR methods, considered impractical due to their reliance on cell division. Furthermore, in the retina of a rat model with retinal degeneration, we successfully performed the direct repair of disease-causing gene mutations using the AAV-mediated HITI method [11]. This intervention resulted in a partial recovery of the vision impairment, highlighting the potential for clinical applications of genome-editing therapy using AAV and genome-editing tools.

The predominant methods for the delivery of adeno-associated virus (AAV) vectors to the heart are intramyocardial injection and intracoronary administration [14,15]. Intramyocardial injection is recognized for its efficacy and direct application; however, it is invasive and can damage cardiac tissue. In contrast, intracoronary administration offers a less invasive, heart-specific alternative but is associated with risks, including the potential for damage to the coronary sinus or veins. Given these drawbacks, there is an urgent need for safer, more effective techniques for gene delivery to the heart. In response to this need, researchers have developed scaffolds, such as hydrogels and fiber mats, integrated with genes, including AAV vectors, for localized and sustained gene delivery to target organs, including the heart [12,16,17]. Such scaffolds support tissue regeneration by facilitating cell attachment, proliferation, and migration [16,18,19]. They can also shield AAV vectors from the immune system. Electrospinning, a process in which a high voltage is applied to polymer solutions to create fine-fiber nonwoven fabrics, has been instrumental in this development. These fine-fiber fabrics, with their extensive large surface areas and extracellular matrix-like structure, offer significant advantages for biomedical applications [20]. The large surface areas of the fabrics allow for the efficient loading and retention of genes, and the utility of electrospun nonwoven fabrics as a substrate for gene delivery has been demonstrated [16,21,22,23].

**Figure 1:**
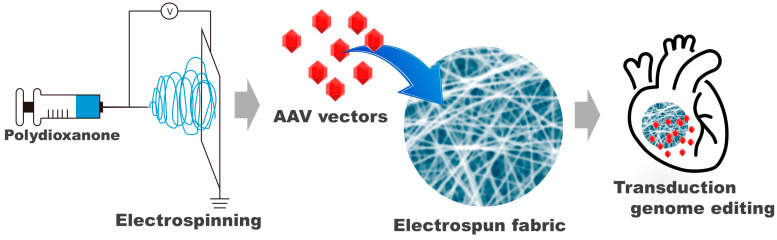
Schematics of fabrication of adeno-associated virus (AAV) vector-loaded electrospun polydioxanone (PDO) nonwoven fabrics and its application to delivery of AAV vectors to the heart.

This study introduces electrospun PDO nonwoven fabrics as a novel medium for AAV vector delivery to the heart and other organs (Figure 1). To the best of our knowledge, while nanoparticles and micelles composed of PDO have been utilized for gene delivery, the use of electrospun PDO nonwoven fabrics for this purpose is unprecedented [1]. We loaded AAV vectors onto the fabrics by immersing them in a solution containing the vectors. The transduction efficiency of these AAV-loaded fabrics was evaluated in vitro by culturing various cell types on them. Additionally, evaluation was conducted by applying them to the mouse liver and heart. The successful implementation of electrospun PDO nonwoven fabrics for AAV vector delivery could have far-reaching implications beyond heart failure treatment. This technology holds promise for the targeted delivery of therapeutic agents in various medical fields.

## 2. Materials and Methods

### 2.1. Materials

Polydioxanone was purchased from Sigma-Aldrich Co., LLC (St. Louis, MO, USA). 1,1,1,3,3,3-hexafluoro-2-propanol (HFP) and 4% paraformaldehyde phosphate-buffered solution (4% PFA) were purchased from FUJIFILM Wako Pure Chemical Co. (Osaka, Japan). Dulbecco’s phosphate-buffered saline (PBS) was purchased from Nacalai Tesque, Inc. (Kyoto, Japan). All reagents were used as received without further purification.

### 2.2. Plasmid

The gRNA cloning plasmid (gRNA_cloning vector), GFP-expression self-complementary AAV plasmid (pscAAV-CAG-GFP), GFP-containing plasmid (pCAG-1BPNLS-Cas9-1BPNLS-2AGFP), Cas9-expression AAV plasmid (pAAV-nEFCas9), and AAV donor backbone plasmid (pAAV-rMERTK-HITI) were obtained from Addgene (Addgene 41824, 83279, 87109, 87115, and 87119, respectively). To construct the myosin heavy chain 6 (Myh6) target gRNA-expression vector (mMyh6-gRNA), we designed the Myh6 target sequence (20 bp target and 3 bp PAM sequence (underlined)) as follows: GCAGCAGAAGATGCACGACGAGG. The Myh6 target was subcloned into the gRNA_cloning vector according to a previously reported protocol (https://media.addgene.org/data/93/40/adf4a4fe-5e77–11e2–9c30–003048dd6500.pdf (20 April 2020, date last accessed)) with the primers 5′- TGGCTTTATATATCTT

GTGGAAAGGACGAAACACCGCAGCAGAAGATGCACGACG-3′ and 5′- GCCTTATTTTAACTTGCTATTTCTAGCTCTAAAACCGTCGTGCATCTTCTGCTGC-3′. To construct a donor/gRNA AAV backbone plasmid (pAAV-Myh6GFP-HITI) for GFP knock-in at the *Myh6* locus, U6-mMyh6gRNA and mMyh6GFP-HITI fragments were amplified from mMyh6-gRNA and pCAG-1BPNLS-Cas9-1BPNLS-2AGFP, respectively, using PrimeSTAR GXL DNA polymerase (Takara Bio, Inc., Shiga, Japan). These PCR fragments were subcloned into pAAV-rMERTK-HITI using an In-fusion HD Cloning Kit (Takara Bio, Inc.).

### 2.3. Cell and Cell Culture

HEK293 cells (American Type Culture Collection, Manassas, VA, USA), human hepatoblastoma-derived HepG2 cells (Riken BRC Cell Bank, Ibaraki, Japan), murine hepatoma-derived Hepa1-6 cells (KAC Co., Ltd., Tokyo, Japan), mouse fibroblasts (10T1/2; Riken BRC Cell Bank), and mouse myoblasts (C2C12; Riken BRC Cell Bank) were cultured in Dulbecco’s modified Eagle’s medium containing 10 *v*/*v*% fetal bovine serum, minimum essential medium non-essential amino acid solution, and penicillin–streptomycin (Thermo Fisher Scientific, Inc., Waltham, MA, USA) at 10 *v*/*v*%, 1 *v*/*v*%, and 1 *v*/*v*%, respectively, in 5% CO_2_ at 37 °C.

### 2.4. Animals

Seven-week-old male Balb/c mice (Oriental Yeast, Tokyo, Japan) were used for gene delivery and genome-editing experiments. They were housed in individual cages in ventilation-controlled rooms with a 12 h light/dark cycle at room temperature. Food pellets and water were provided ad libitum. All animal experiments were conducted in accordance with the guidelines of the Osaka University Animal Care and Use Committee and were approved by the committee. Efforts were made to minimize the number of animals used and their suffering.

### 2.5. Production of AAV Vectors

AAV vectors were produced in AAVpro 293T cells (Takara Bio, Inc., Shiga, Japan) through the co-transfection of helper, capsid-expression, and vector plasmids. The pAD5 (a helper plasmid) and pXR-DJ (an AAV-DJ capsid-expression plasmid) were used for packaging all AAV vectors. For the preparation of GFP-expression self-complementary AAV (scAAV-GFP), a co-transfection was performed using pAD5, pXR-DJ, and pscAAV-CAG-GFP. For the preparation of Cas9-expression AAV (AAV-Cas9), a co-transfection was performed using pAD5, pXR-DJ, and pAAV-nEFCas9 into AAVpro 293T cells. For the preparation of gRNA expression with knock-in donor AAV (AAV-Myh6GFP-HITI), a co-transfection was performed using pAD5, pXR-DJ, and pAAV-Myh6GFP-HITI. For the co-transfection, fresh medium was added 1 h before transfection. In 10 × 15 cm dishes, nearly confluent cells were transfected with 10 μg each of the three plasmids using PEIMax (Polyscience, Inc., Warrington, PA, USA) at a 5:1 ratio. After incubating the mixtures for 15 min at room temperature, they were added to the cells. At 3 days post-transfection, vectors were purified with the AAVpro Purification Kit and titrated using the AAVpro Titration Kit (Takara Bio, Inc., Shiga, Japan).

### 2.6. Electrpspinnig of PDO Fibers

PDO nonwoven fabrics were electrospun from a 16.8 w/v% PDO solution in HFP, as previously reported [4], using a 5 mL syringe with an 18-gauge needle, at a flow rate of 1.0 mL/h and an 18 kV voltage. The setup, with a 12 cm distance between the needle and collector, was operated by a Kato Tech electrospinning unit. The resultant fabrics were cut into rectangles (7 mm × 10 mm) for subsequent experiments. Fiber diameters in the fibers were determined from SEM images using ImageJ software (Version 1.53f, NIH, Bethesda, MD, USA).

### 2.7. Loading GFP-Expression AAV Vectors onto Electrospun PDO Nonwoven Fabrics

A 70 μL solution containing scAAV-GFP vectors with various titers was absorbed uniformly onto a rectangle PDO nonwoven fabric (7 mm × 10 mm). After 10 min of standing, the fabric was rinsed with PBS.

### 2.8. AAV Release from PDO Fabrics

The release of the AAV from the PDO fabrics, obtained by bringing the rectangular fabrics into contact with a solution containing 2.0 × 10^7^ genome copies (GCs) of scAAV-GFP, was determined by measuring the AAV titer in the culture medium supernatant following the pre-incubation of the PDO fabrics at 37 °C. The supernatants were collected at 2, 4, 6, 8, and 30 h post-incubation. Subsequently, HEK293 cells, cultured for 3 days in these supernatants, were assessed for GFP expression via flow cytometry. 

### 2.9. Transduction to Cultured Cells

The effect of the loaded amount of AAV vectors onto the fabrics on the transduction efficiency was evaluated using HEK293 cells. The cells were seeded at 1.4 × 10^3^ cells/mm^2^-fabric on the rectangular nonwoven fabrics contacted with a solution containing 2.0 × 10^5–8^ GCs of scAAV-GFP. The GFP expression of the cells was measured by fluorescence microscopy and flow cytometry at 5 days of culture. The cells for the flow cytometry were obtained by the trypsin treatment of the cells on the fabrics.

The transduction to cultured cells was also conducted for 10T1/2, Hepa1-6, HepG2, and C2C12 cells using the fabrics contacted with a solution containing 2.0 × 10^7^ GCs of scAAV-GFP.

### 2.10. Transduction and Genome Editing In Vivo 

To evaluate the transduction to the liver, the rectangular fabrics were contacted with a solution containing 1.0 × 10^10^ GCs of scAAV-GFP vectors. Mice were anesthetized with isoflurane, and following a skin incision, the liver was exposed. The fabrics, with AAV vectors (three animals) or without (two animals), were affixed to the liver, after which the skin was sutured back. After 7 days of the surgery, the mice were deeply anesthetized with isoflurane and trans-cardially perfused with PBS followed by 4% PFA.

To assess the heart transduction and genome editing, the rectangular fabrics were contacted with a solution containing 1.0 × 10^10^ GCs of scAAV-GFP vectors or a combination of 1.0 × 10^10^ GCs each of AAV-Cas9 and AAV-Myh6GFP-HITI vectors. Mice were anesthetized with isoflurane, and following a skin incision, the heart was exposed. The fabrics, with AAV vectors (three animals) or without (two animals), were affixed to the heart, after which the skin was sutured back. After 7 or 14 days of the surgery, the mice were deeply anesthetized with isoflurane and trans-cardially perfused with PBS followed by 4% PFA.

### 2.11. Immunostaining 

Extracted tissues were fixed, sucrose-protected, embedded in O.C.T., and sectioned at 10 μm on a cryostat (Reichart-Jung, Inc., Heidelberg, Germany) at −20 °C. Sections were stained using chicken anti-GFP (1:200, Aves labs, Davis, CA, USA), followed by anti-chicken Alexa Fluor 488 secondary antibody (1:500, Thermo Fisher Scientific, Inc., Waltham, MA, USA) and Hoechst 33342 (1:2000, Thermo Fisher Scientific, Inc. Waltham, MA, USA), after permeabilization and blocking with normal goat serum (Jackson Immuno Research Laboratories, Inc., West Grove, PA, USA) and Triton X-100. Sections were mounted post-PBS washes.

## 3. Results and Discussion 

### 3.1. Release of AAV Vectors from PDO Nonwoven Fabrics

**Figure 2:**
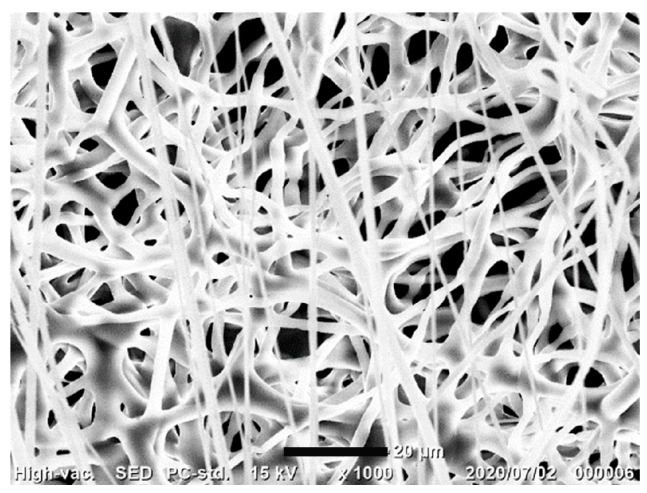
A scanning electron microscope image of electrospun PDO nonwoven fabric.

**Figure 3:**
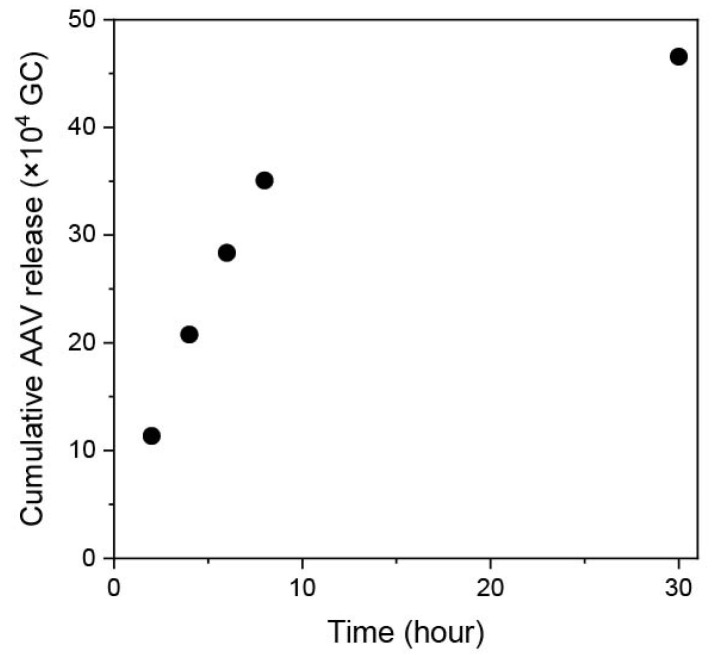
Release profile of AAV vectors from PDO electrospun nonwoven fabrics. Cumulative release of the vectors from the fabrics obtained by soaking in a solution containing 2.0 × 10^7^ GCs of scAAV-GFP vectors. Data represent means ± SDs.

Figure 2 shows an electrospun PDO nonwoven fabric obtained from HFP dissolving PDO. The average diameter of the fibers was 2.38 ± 0.77 μm. To confirm the release of AAV vectors from the nonwoven fabrics with AAV vectors, the transduction ability of the vectors in the supernatant was evaluated. As shown in Figure 3, AAV vectors loaded onto the PDO fabrics continued to be released during the 30 h test. The release of the vectors could be due to the cleavage of hydrogen bonding as well as the hydrophobic interactions between the vectors and PDO fibers [24], or to the hydrolytic degradation of the fabrics; this is consistent with previous studies for PDO-based constructs [25,26]. It is important to note that the release profile observed in this study may differ from those in in vivo conditions due to environmental differences, such as the pH and the presence of proteins in the body. Nevertheless, these initial results regarding the AAV vector release from PDO fabrics have led us to conduct further experiments. 

### 3.2. In Vitro Transcudtion

**Figure 4:**
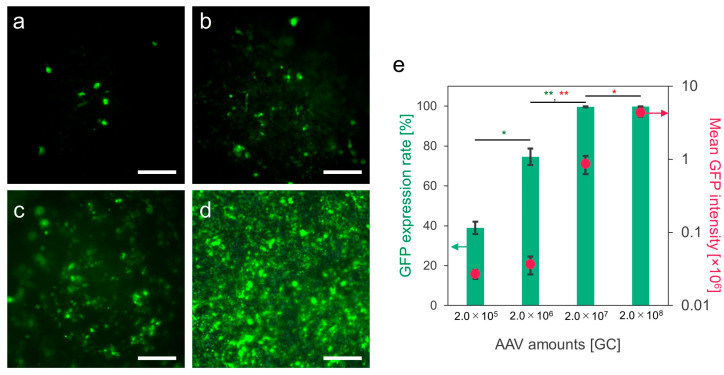
Transduction to HEK293 cells on PDO nonwoven fabrics with scAAV-GFP vectors. Fluorescence images of the transduced cells on the fabrics (green) with the vectors obtained by soaking in the solutions containing (**a**) 2.0 × 10^5^, (**b**) 2.0 × 10^6^, (**c**) 2.0 × 10^7^, and (**d**) 2.0 × 10^8^ GCs of the vectors. Scale bars represent 200 μm. (**e**) GFP expression rates were calculated from the number of cells expressing GFP, along with the mean GFP intensity. Data represent means ± SDs. * *p* < 0.05, ** *p* < 0.01.

The transduction ability of the scAAV-GFP vectors loaded onto the PDO nonwoven fabrics was first evaluated by culturing HEK293 cells for 5 days on the fabrics obtained by treatment with the solutions containing 2.0 × 10^5^, 2.0 × 10^6^, 2.0 × 10^7^, and 2.0 × 10^8^ GCs of scAAV-GFP vectors. The GFP expression rate increased with the increasing content of the vectors in the treatment solutions (Figure 4a–d). Almost all the cells expressed GFP on the fabrics obtained by the treatment with the solutions containing 2.0 × 10^7^ and 2.0 × 10^8^ GC scAAV-GFP vectors (Figure 4e). Despite the comparable GFP expression rates on these fabrics (99.7 ± 0.2% and 99.8 ± 0.1%), the mean fluorescence intensity, indicative of the amount of GFP expression in each cell, was approximately five times higher on the fabrics treated with the solution containing the 2.0 × 10^8^ GC scAAV-GFP vectors compared to the value observed for the cells on the fabrics treated with the solution containing the 2.0 × 10^7^ GC vectors (Figure 4e). The higher expression of GFP means the greater amount of scAAV-GFP vectors introduced into each cell. An important finding is that the transduction ability per fabric can be adjusted by the content of AAV vectors in the treatment solutions. This observation is not unique to electrospun PDO nonwoven fabrics; a similar result was reported for alginate hydrogels enclosing AAV vectors [19]. 

**Figure 5:**
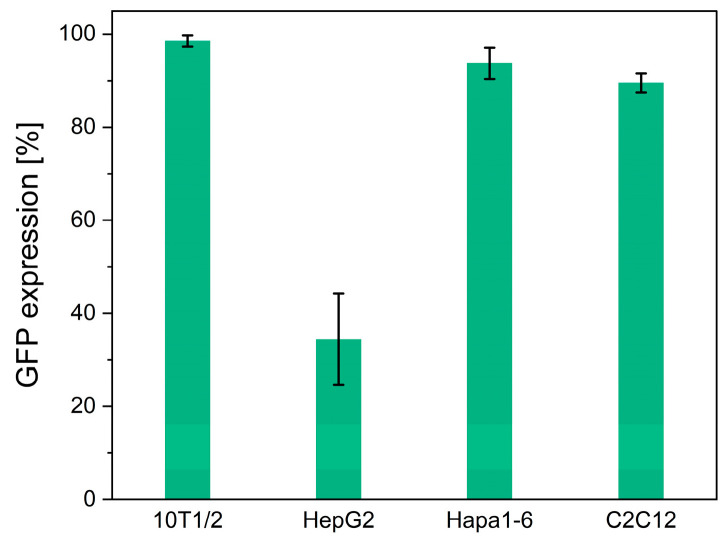
Transduction to 10T1/2 cells, HepG2 cells, Hepa1-6 cells, and C2C12 cells on PDO electrospun nonwoven fabrics with AAV vectors. Fluorescence images of the transduced cells on the nonwoven fabrics. GFP expression rates are calculated from the number of GFP-positive cells. Data represent means ± SDs.

The versatility of the transduction to various cell types was investigated by culturing mouse fibroblast 10T1/2, human hepatoma HepG2, mouse hepatoma Hepa1-6, and mouse muscle C2C12 cells for 5 days on the PDO fabrics obtained by the treatment with the solution containing 2.0 × 10^7^ GCs of scAAV-GFP vectors. All the cells expressed GFP (Figure 5). The GFP expression rates of the 10T1/2, HepG2, Hepa1-6, and C2C12 cells were 98.5 ± 1.2%, 34.4 ± 9.8%, 93.7 ± 3.3%, and 89.5 ± 2.0%, respectively (Figure 5). These results demonstrate the potential of electrospun PDO fabrics as a versatile platform for AAV vector delivery to various cell types. The difference in the degree of transduction, typically indicated by the lowest GFP expression in HepG2 cells, does not pose a problem in our system. The transduction efficiency of AAV vectors varies significantly between cell types due to factors such as the cell surface receptor expression, serotype specificity, and promoter activity [27,28,29]. It is out of the scope of this study, but modifications to the AAV capsid or the use of alternative serotypes with different receptor specificities could improve the transduction rates in HepG2 cells [27,28,29].

### 3.3. In Vivo Transduction and Genome Editing

**Figure 6:**
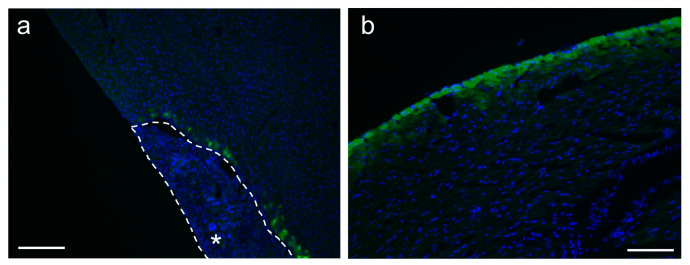
Transduction to mouse tissues with attached electrospun PDO nonwoven fabrics with scAAV-GFP vectors. Representative images of immunostaining of GFP-positive cells (green) with nucleic staining by Hoechst 33342 (blue) of the (**a**) liver and (**b**) heart, 7 days after their attachment. The scale bars represent 200 μm. * panel (**a**): PDO fabric adhering to the liver surface.

We evaluated the potential of the electrospun PDO fabrics loaded with AAV vectors for transduction in vivo by applying the fabrics loading 1.0 × 10^10^ GCs of scAAV-GFP vectors onto the liver and heart of the mouse for 7 days. As typically shown in Figure 6, the PDO fabrics remained attached to the surfaces of both the liver and heart throughout the duration, and green fluorescence, indicative of GFP expression, was observed on the surfaces of both organs. Notably, a higher expression of GFP was detected in the areas surrounding the fabric application (Figure 6a). These findings suggest that AAV vector-loaded electrospun PDO nonwoven fabrics are effective for delivering these vectors to targeted sites. The retention of these fabrics on the organ surfaces after 7 days can be attributed to the absence of fluid flow over these surfaces that could potentially cause detachment, and to the small fiber diameters of the fabrics, which facilitated their adsorption onto the organ surfaces. We reported a similar result for electrospun gelatin fabrics applied to mouse liver [23]. As reported in various studies, electrospun fibers have been shown to be effective for the local delivery of vectors [16,21,22,23,30]. However, it is impossible to completely prevent their off-target distribution, and not only with PDO fibers, due to the diffusion of the released vectors through the bloodstream and body fluids. Coating the surface of the fabrics placed on the target tissue with an additional material, such as a genetic material-free nonwoven fabric or hydrogel, would further suppress off-target distribution.

**Figure 7:**
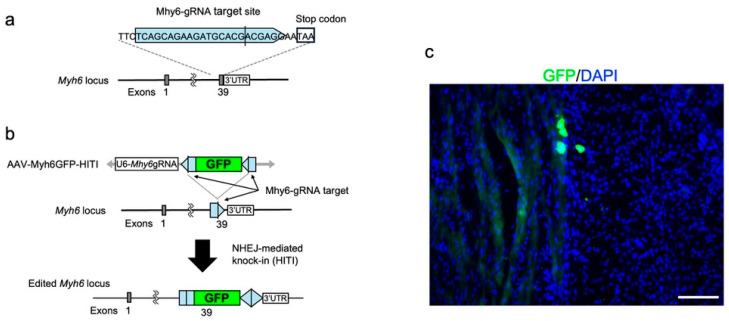
Genome editing in mouse heart with attached electrospun PDO nonwoven fabrics with AAV vectors for gene knock-in through HITI. Schematic representation of (**a**) the Cas9/gRNA target site at exon 39 of the *Myh6* locus and (**b**) genome-editing assays for GFP knock-in through HITI. The light-blue pentagon represents the Cas9/gRNA target sequence. The black line within the light-blue pentagon represents the Cas9 cleavage site. (**c**) A representative image of immunostaining of GFP-positive cells (green) with nucleic staining by Hoechst 33342 (blue), 14 days after the attachment of the fabrics to the heart below the fabrics with the AAV vectors for gene knock-in through HITI. The scale bar represents 200 μm.

The potential for genome editing in heart tissue using electrospun PDO fabrics was also investigated. The HITI-mediated targeted GFP knock-in at the *Myh6* gene, which is highly expressed in cardiomyocytes, was evaluated based on the GFP expression in the heart tissues following genome-editing treatment. HITI is mediated by NHEJ, which is active in non-dividing cells, resulting in a gene knock-in in non-dividing tissues, including the heart [11]. We designed AAV-mediated HITI constructs intended to incorporate a GFP cassette downstream of the *Myh6* gene. This design aims to induce the expression of a MYH6-GFP fusion protein in the cardiomyocytes (Figure 7a). To evaluate gene knock-in via HITI, we simultaneously delivered 1.0 × 10^10^ GCs of Cas9-expression AAV (AAV-Cas9) and gRNA expression with knock-in donor AAV (AAV-Myh6GFP-HITI) vectors. The AAV mixture was loaded onto the PDO fabrics, which were subsequently affixed to the heart. As a result, a subset of cardiomyocytes expressed GFP driven by *Myh6* through HITI (Figure 7b). The use of PDO nonwoven fabrics with these AAV-mediated HITI vectors facilitated gene knock-in in the heart, aligning with the results observed with the intravenous injection of AAV carrying genome-editing components [11]. Therefore, PDO electrospun nonwoven fabrics with AAV vectors have the potential for gene therapy for cardiac diseases using genome editing.

### 3.4. Limitations and Future Research Directions

Although the above results are preliminary for clinical applications, they demonstrate the potential of electrospun PDO nonwoven fabrics loaded with AAV vectors for cardiac transduction and genome editing. Future research will need to explore several approaches to improve the transduction efficiency of the fabrics. 

First, it is critical to investigate the effect of the fiber diameter within the fabrics. In this study, the fibers had an average diameter of 2.38 ± 0.77 μm. Reducing the fiber diameter while maintaining the same fiber density increases the surface area-to-volume ratio, which increases the amount of AAV vectors loaded by the fabrics. Because the fiber diameter affects the rate of fiber degradation [31], controlling the fiber diameter may control the release of vectors. The diameter of the electrospun fibers can be adjusted by modifying electrospinning parameters such as the polymer concentration, applied voltage, and flow rate [32,33]. Adjusting the number of vectors loaded and the degradation rates of the fabrics could lead to an improved transduction efficiency. 

Second, the choice of the AAV vector serotype plays a significant role in the transduction efficiency. In this study, we used AAV-DJ vectors, which were transduced to various tissues. Three serotypes of AAV vectors, the AAV1, AAV8, and AAV9 vectors, have high transduction efficiencies to the heart [34]. AAV9 vectors especially achieved efficient transduction to cardiomyocytes through systemic injection [35]. AAV-DJ vectors may be loaded onto the PDO nonwoven fabrics through hydrogen bonding and hydrolytic interaction, and PDO nonwoven fabrics can load other serotypes of AAV vectors. Therefore, the PDO nonwoven fabrics were applied to the other serotypes of AAV vectors, resulting in the possibility of a higher transduction efficiency to the heart. 

Finally, the in vivo experimental setup can influence the results. In this study, the in vivo gene delivery was evaluated for 1 or 2 weeks. The in vivo degradation timeline of the fabrics was not evaluated. Prolonged attachment of the fabrics may enhance the transduction efficiency. 

Given these considerations, determining the optimal conditions for cardiac gene therapy remains challenging. Nevertheless, future investigations into these aspects are essential for the advancement of cardiac gene therapy strategies. By addressing these areas, future research can build on the foundational work presented here and potentially unlock more effective gene therapy techniques for the treatment of cardiac diseases and beyond.

## 4. Conclusions

In this study, we aimed to demonstrate the feasibility and efficiency of using electrospun PDO nonwoven fabrics for AAV vector delivery to organs by adhering them to organ surfaces, providing potency for cardiac gene therapy. The results of this study indicate that these fabrics can significantly enhance the AAV vector delivery and expression in targeted tissues both in vitro and in vivo through transduction and genome editing. Specifically, we observed nearly universal GFP expression in vitro on fabrics treated with the highest vector concentrations, and remarkable GFP expression in vivo on the surfaces of mouse liver and heart tissues. These outcomes highlight the potential of electrospun PDO nonwoven fabrics in medical applications, particularly for heart failure treatment. Future research will focus on optimizing the electrospinning process to increase the vector-loading efficiency, investigating the effect of AAV vector serotypes on the transduction efficiency, and conducting detailed in vivo studies to determine the optimal conditions for cardiac gene therapy. This study lays the groundwork for the advancement of gene delivery techniques and offers a novel approach to the treatment of heart diseases and potentially other diseases requiring targeted gene therapy.

## Data Availability

Dataset available on request from the authors.
